# Post-translational modifications play indispensable roles in cold stress responses of plants

**DOI:** 10.3389/fpls.2026.1880143

**Published:** 2026-07-23

**Authors:** Mingfeng Zhao, Liang Chen, Suiwen Hou

**Affiliations:** Key Laboratory of Gene Editing for Breeding, Key Laboratory of Cell Activities and Stress Adaptations, Gansu Province Ministry of Education, School of Life Sciences, Lanzhou University, Lanzhou, Gansu Province, China

**Keywords:** cold stress, physiological responses, post-translational modifications (PTMs), signal transduction, temperature responses

## Abstract

Cold environments significantly influence plant growth, development, geographical distribution, and yield. A growing body of research has focused on elucidating the molecular mechanisms underlying plant responses to cold stress, among which post-translational modifications (PTMs) of proteins play a pivotal role by regulating protein stability, activity, and protein-protein interactions. In this review, we summarize regulatory mechanisms of several types of PTMs, including phosphorylation, ubiquitination, SUMOylation, acetylation, crotonylation, and S-acylation, in the cold stress signaling pathway. These dynamic modifications allow plants to mount precise and adaptive responses, effectively balance defense with developmental processes, and establish a molecular foundation for adaptation to cold environments. A comprehensive understanding of the cascading relationships and collaborative regulatory networks among PTMs is indispensable for deciphering the molecular logic of plant cold adaptation. Moreover, further exploration of the temporal and spatial dynamics of these modifications and their functional associations will continue to refine our mechanistic understanding of the regulatory networks governing plant cold tolerance.

## 1 Introduction

Cold stress profoundly impairs plant growth, development, geographical distribution, and yields (Ritonga and Chen, 2020). Throughout their long-term evolution, plants have evolved a range of defense strategies and adaptive mechanisms at the physiological, biochemical, and molecular levels to withstand environmental cold stress. The mechanisms underlying plant tolerance to cold stress have been extensively elucidated in *Arabidopsis*. Three *C-REPEAT BINDING FACTOR/DRE BINDING FACTOR1 (CBF/DREB1)* transcription factors play redundant and core roles in cold and freezing stress tolerance (Thomashow, 1999; Novillo et al., 2007; Jia et al., 2016; Zhao et al., 2016). These transcription factors are rapidly and strongly induced upon exposure to low temperatures, subsequently activating the expression of *COLD-REGULATED (COR)* genes, which leads to the accumulation of protective substances such as osmolytes and cryoprotective proteins that facilitate cold or freezing tolerance (Thomashow, 1999; Shi et al., 2018; Ding et al., 2020). The identification of the cold sensor COLD1 in rice further advanced our understanding of how plants perceive low-temperature signals at the plasma membrane (Ma et al., 2015).

Post-translational modifications (PTMs) of proteins in which a small molecule is added to modify the localization, stability, or function of a target protein are critical for cell survival under acute stress conditions due to their rapid reversibility and responsiveness to environmental changes (Liu et al., 2019). To date, more than 400 types of PTMs have been identified. It is estimated that 5% of the proteome consists of enzymes involved in PTMs, including kinases, phosphatases, transferases, and ligases (Ramazi and Zahiri, 2021). The last decade has seen rapid progress in the study of plant PTMs, with improved analytical approaches and further elucidation of their functions and regulatory mechanisms in plants. In addition, the regulatory roles of PTMs in plant stress responses, including their crosstalk and coordination, have been comprehensively summarized (Li et al., 2025). Recent comprehensive reviews have systematically outlined cold signal transduction pathways, including cold perception, Ca^2+^ signaling, MITOGEN-ACTIVATED PROTEIN KINASE (MAPK) cascades, the canonical INDUCER OF CBF EXPRESSION 1 (ICE1)-CBF-COR module (Qian et al., 2024). It has been demonstrated that PTMs including phosphorylation, ubiquitination, SUMOylation, lysine crotonylation, acetylation and S-acylation play essential roles in plant cold stress responses. In this review, we systematically summarize recent advances in elucidating the regulatory mechanisms of these PTMs in cold signaling pathways. We also discuss conserved and species-specific PTM regulatory modules, the complex crosstalk among distinct PTM types, and the translational potential of PTM knowledge for crop improvement.

## 2 Phosphorylation

Protein phosphorylation is mediated by kinases and phosphatases, which enable the reversible transfer of the γ-phosphate group from adenosine triphosphate (ATP) to specific amino acid residues (Ser, Thr, or Tyr) on substrate proteins, thereby modifying their activity (Stone and Walker, 1995). Protein phosphorylation plays a critical role in signaling for plant development and acclimation to environmental challenges by precisely phosphorylating key components within the signaling cascades (Chen et al., 2021; Zhang et al., 2023).

Different kinases exert distinct regulatory effects on the cold signaling pathway. For instance, OPEN STOMATA 1 (OST1)/SnRK2.6/SnRK2E protein kinase, CALCIUM-DEPENDENT PROTEIN KINASE 28 (CPK28), CPK3, and PLANT PEPTIDE CONTAINING SULFATED TYROSINE1 RECEPTOR (PSY1R) positively regulate cold resistance. In contrast, BRASSINOSTEROIDS INSENSITIVE 2 (BIN2) negatively regulates cold resistance by phosphorylating ICE1. Additionally, CALCIUM/CALMODULIN-REGULATED RECEPTOR-LIKE KINASE 1 (CRLK1) and COLD RESPONSE PROTEIN KINASE 1 (CRPK1) negatively modulate cold signaling. Furthermore, the MAPK cascade exhibits species-specific roles: it negatively regulates cold responses in *Arabidopsis* and maize, but positively regulates them in rice (**Table 1**).

**Table 1:** The main proteins undergoing PTMs modification in the cold signaling pathway.

Name	Function	References
OST1	Phosphorylate ICE1 and enhance its stability	(Ding et al., 2015)
CPK28	Phosphorylation of NLP7 causes it to move from the cytoplasm to the nucleus.	(Ding et al., 2022)
ZmCPK17	Phosphorylation of COOL1, negatively regulating cold resistance	(Zeng et al., 2025)
PSY1R	Enhance the activity of CNGC20 by phosphorylation	(Peng et al., 2024)
CRPK1	Phosphorylates CNGC20, thereby facilitating its degradation.	(Peng et al., 2024)
CPK3	Activate CNGC5/6 to trigger Ca^2+^ influx.	(Ming et al., 2025)
MPK3/6	Promote the degradation of ICE1	(Zhao et al., 2017)
MPK4	Inhibit the activity of MPK3 positively regulates cold resistance	(Zhao et al., 2017)
ZmMPK8	Promote bZIP68 stability and degradation of ZmRR1	(Zeng et al., 2021)
BIN2	Promote the degradation of ICE1 in the later stage of cold stress	(Ye et al., 2019)
PP2CG1	inhibit the kinase activity of OST1	(Lv et al., 2021)
PUB25/26	Polyubiquitinate ICE1 and MYB15	(Wang et al., 2023b)
HOS1	Promote ICE1 degradation by ubiquitination.	(Ding et al., 2015)
AtATL78	negatively regulating cold resistance	(Kim and Kim, 2013)
EBF1/2	Promote the degradation of PIF3	(Jiang et al., 2017)
MAX2	Enhancing cold tolerance by inhibiting WRKY41	(Wang et al., 2023a)
COP1	Promote the degradation of HY5	(Li et al., 2021)
SIZ	SUMOylate ICE1 and enhance its stability	(Miura et al., 2007)
HD2C	Promote the deacetylation of COR	(Park et al., 2018)
OsHDA716	Promote the deacetylation of OsbZIP46	(Sun et al., 2024)
MtAPT1	Promote the de-S-acylation of MtNAC80	(Ye et al., 2024)

### 2.1 Phosphorylation triggered by Ca^2+^ signals regulates plant cold responses

**Figure 1:**
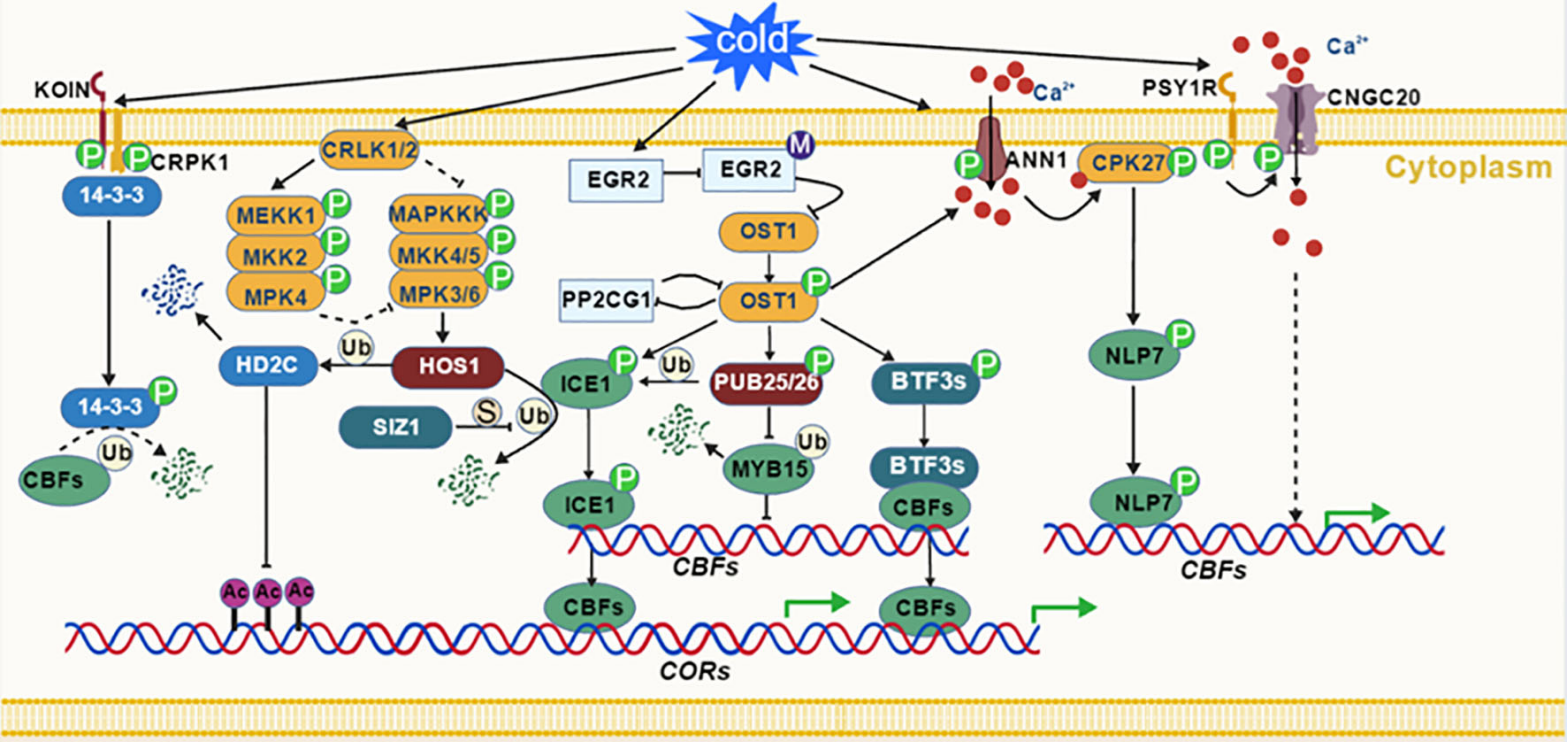
The role of PTMs and their crosstalk in regulation of cold signal transduction in *Arabidopsis*. When plants sense cold stress, the accumulation of unprocessed EGR2 reduces the interaction between EGR2 and OST1, thereby activating OST1. The activated OST1 phosphorylates AtANN1, enhancing its binding activity with calcium ions, leading to an increase in intracellular calcium ion concentration. Additionally, cold exposure activates PSY1R, which phosphorylates and enhances the activity of CNGC20, further increasing the intracellular Ca^2+^ level. CPK28 is activated upon sensing Ca^2+^ and phosphorylates NLP7, promoting the transfer of NLP7 from the cytoplasm to the nucleus, and activating the expression of downstream COR genes. Ca^2+^ also activate CRLK1/2, which enhances the MEKK1-MKK2-MPK4 signaling cascade reaction and inhibits MAPK3/6. MAPK3/6 phosphorylates ICE1, and then ICE1 is ubiquitinated and degraded by the HOS1 protein, thereby limiting the activation of CBF mediated by ICE1. Therefore, CRLK1/2 enhances cold tolerance by promoting the activation of CBF mediated by ICE1. Moreover, the cold-activated OST1 phosphorylates ICE1 and inhibits the ubiquitination and degradation process of ICE1 mediated by HOS1, thereby improving the cold tolerance of plants. SIZ performs SUMO modification on ICE1, inhibiting the ubiquitination and degradation process of ICE1 caused by HOS1, thereby enhancing cold tolerance. OST1 phosphorylates PUB25/26, which then ubiquitinates ICE1 and ubiquitinates the cold inhibitory factor MYB15, resulting in the stabilization of ICE1 and the degradation of MYB15, thereby promoting the cold tolerance of plants. OST1 also phosphorylates the BTF3 protein, promoting the formation of the BTF3-CBF complex, thereby stabilizing CBF proteins and enhancing the transcription of CBF genes. OST1 further phosphorylates PP2CG1 to inhibit its phosphatase activity, releasing a more active OST1, thereby forming a feedback loop in cold signal transduction. Moreover, the cold-activated CRPK1 phosphorylates KOIN, and the KOIN-CRPK1 complex subsequently phosphorylates the 14-3–3 protein, promoting the transfer of 14-3–3 protein to the nucleus and helping to degrade CBF transcription factors through the ubiquitin pathway. Ubiquitination and SUMOylation antagonistically regulate ICE1 stability at the CBF hub. OST1 phosphorylation activates ICE1, whereas MPK3/6 phosphorylation promotes its degradation. Meanwhile, HOS15-mediated HD2C degradation increases COR promoter acetylation and chromatin accessibility, linking ubiquitination to epigenetic regulation. Multiple types of post-translational modifications (PTMs) extensively crosstalk with one another to jointly govern all tiers of cold signaling cascades, from plasma membrane cold sensors to nuclear CBF-COR transcriptional programs. P, phosphorylation; Ub, ubiquitination; S, SUMOylation; Ac, histone acetylation; M, myristoylation.

Under ambient conditions, the concentration of Ca^2+^ in the cytosol ([Ca^2+^] cyt) is maintained at a low steady-state level, resulting in low basal expression levels of *CBFs* and their target genes. Cold stress induces the opening of unknown Ca^2+^ channels, leading to a rapid increase in cytosolic Ca^2+^ concentration. CPK28, a CPK family member which harbors four EF-hand Ca^2+^ -binding motifs and a Ser/Thr kinase domain that brings together both Ca^2+^ sensing and responding activity within a single protein (Chen et al., 2021), rapidly detects and responds to the Ca^2+^ signals, becoming activated. Once activated, CPK28 phosphorylates five residues of the transcription factor NIN-LIKE PROTEIN 7 (NLP7) (Ser783, Ser793, Ser807, Ser808, and Thr817) in a Ca^2+^ -dependent manner. This phosphorylation event triggers the translocation of NLP7 from the cytoplasm to the nucleus where it initiates transcriptional reprogramming that enhances freeze resistance in *Arabidopsis* (**Figure 1**) (Ding et al., 2022). Recently, ZmCPK17 was shown to phosphorylate the bHLH transcription factor *COLD-RESPONSIVE OPERATION LOCUS 1* (*COOL1*), a key regulator of cold tolerance in maize, and inhibit COOL1 ubiquitination and degradation. Stabilized COOL1 subsequently represses downstream cold-responsive genes, contributing to the regulation of cold tolerance (Zeng et al., 2025).

The RECEPTOR-LIKE KINASES (RLKs), including PSY1R, CPK3 and the receptor-like cytoplasmic kinase CRPK1, are also activated under cold stress. PSY1R phosphorylates the Ca^2+^-permeable channels CYCLIC NUCLEOTIDE-GATED CHANNEL20 (CNGC20) and enhances its channel activity, thereby elevating [Ca^2+^]cyt. CNGC20 also undergoes CRPK1-mediated phosphorylation and is degraded via the 26S proteasome pathway during prolonged cold treatment, leading to attenuated cold response in *Arabidopsis* (**Figure 1**) (Peng et al., 2024). CPK3 activates CNGC5/6 to trigger Ca^2+^ influx. Subsequently, Ca^2+^ interacts with CALMODULIN 2 (CaM2), which negatively regulates the activity of CNGC channels, terminates the signal transduction, and thus maintains the steady state of cytosolic Ca^2+^ homeostasis (Ming et al., 2025). CRPK1 is recruited by the pseudo-kinase KINASE ON THE INSIDE (KOIN), which is localized at the plasma membrane and acts as a platform for signal sensing and integration under low-temperature stress. Activated CRPK1 phosphorylates KOIN, promoting its recycling from endosomes to the plasma membrane to maintain the stability of the signaling platform. Concurrently, CRPK1 phosphorylates the 14-3–3 proteins, facilitating their nuclear translocation into the nucleus where they promote ubiquitination and degradation of CBFs. The reduction in CBF levels ultimately leads to downregulation of the key developmental regulator *SHORT-ROOT* (*SHR*), which inhibits root cell division and elongation and thus causes the plant to enter an energy-saving “hibernation” state to adapt to the cold environment (**Figure 1**) (Liu et al., 2017; Zhang et al., 2025).

### 2.2 OST1-mediated phosphorylation amplifies cold signals through multi-target regulation

OST1 was first identified as a Ser/Thr protein kinase involved in abscisic acid (ABA) signaling (Mustilli et al., 2002). The accumulation of ABA under stress leads to its binding to the receptor PYR/PYL/RCAR and subsequently to the PROTEIN PHOSPHATASE 2C (PP2C), releasing OST1 from PP2C-mediated inhibition (Ma et al., 2009; Park et al., 2009; Hao et al., 2011). Activated OST1 has been demonstrated to phosphorylate ABRE-binding proteins/factors (AREBs/ABFs) which regulate stress-responsive genes and the anion/cation channels SLOW ANION CHANNEL 1 (SLAC 1) and POTASSIUM CHANNEL 1 which modulate stomatal opening and closure (Furihata et al., 2006). Under cold stress, OST1 is activated by auto-phosphorylation, which is governed by the protein phosphatase CLADE E GROWTH-REGULATING 2 (EGR2). OST1 promotes the transcriptional activity of ICE1 by phosphorylating ICE1, an MYC-like basic-helix-loop-helix (bHLH) transcription factor. Concurrently, OST1-mediated ICE1 phosphorylation inhibits the interaction between ICE1 and the E3 ligase HIGH EXPRESSION OF OSMOTICALLY RESPONSIVE GENE1 (HOS1) which degrades ICE1 via 26S proteasomal degradation, thus enhancing ICE1 stability. Activated and stabilized ICE1 functions as a master switch to control *CBF* expression levels, thereby promoting freezing tolerance (**Figure 1**) (Ding et al., 2015).

Low temperatures also facilitate OST1 activity by disrupting the interaction between OST1 and PP2CG1 which inhibits OST1 by dephosphorylating OST1 at Ser29, Ser171, Ser175 and Thr176. Interestingly, OST1 in turn phosphorylates PP2CG1 at Ser365 to inhibit its phosphatase activity, constituting a positive feedback loop to enhance OST1 activity in response to cold stress (Lv et al., 2021). Moreover, OST1 phosphorylates the E3 ubiquitin ligases PLANT U-BOX 25 and 26 (PUB25 and PUB26) at Thr95 and Thr94, respectively. This modification promotes PUB25/PUB26-mediated ubiquitination and degradation of their downstream target, the transcription factor MYB15 which inhibits *CBF* expression, thereby enhancing the cold resistance of plants. OST1 also targets the Ca^2+^-permeable transporter ANNEXIN 1 (AtANN1) under cold stress. Specifically, OST1 phosphorylates AtANN1 at Ser289, which potentiates its Ca^2+^ binding affinity and transport activity. This results in an increase in cytoplasmic Ca^2+^ concentration under cold stress, which subsequently promotes CBF and COR expression and positively regulates freezing tolerance (**Figure 1**) (Liu et al., 2021).

BASIC TRANSCRIPTION FACTOR 3 (BTF3) and its homolog BTF3-LIKE PROTEIN (BTF3L) serve as β-subunits of the NASCENT POLYPEPTIDE-ASSOCIATED COMPLEX (NAC). The NAC functions as an initial factor that interacts reversibly with nascent polypeptides as they emerge from the ribosome exit tunnel, preventing them from binding inadvertently to other cytosolic factors. Under cold stress, activated OST1 phosphorylates BTF3L at Ser50, which on the one hand enhances the interaction of the BTF3L-CBF complex to stabilize CBF proteins, and on the other hand indirectly promotes the transcription of *CBF* genes. This double action of BTF3L-CBF protein leads to upregulation of *CBF* target genes, thus enhancing freezing resistance (**Figure 1**) (Ding et al., 2018).

### 2.3 Brassinosteroid signaling regulates cold tolerance through BIN2

**Figure 2:**
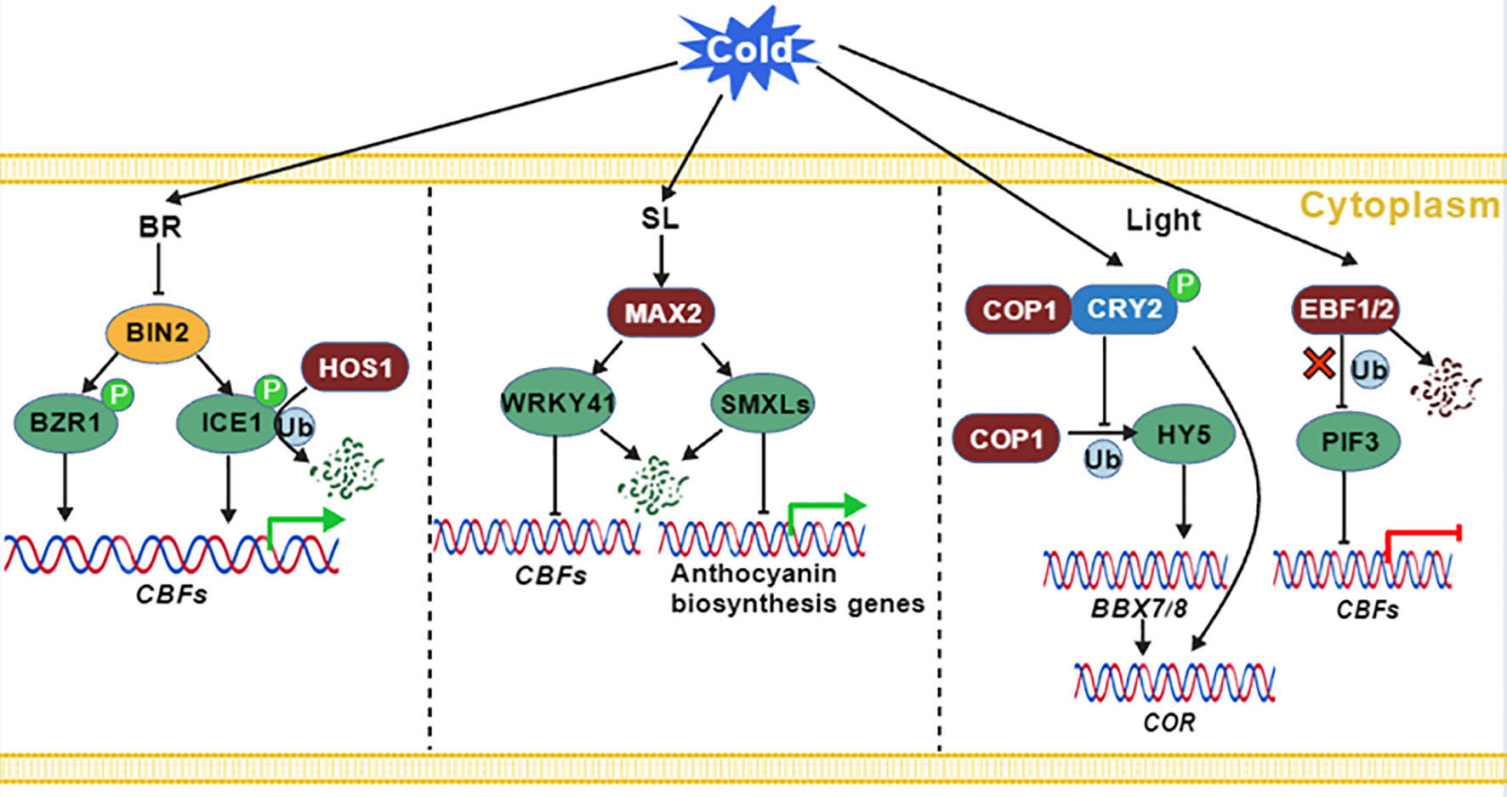
PTM mediated crosstalk between cold signaling and other signaling in *Arabidopsis*. The BZR1 and BES1 proteins in the BR signaling pathway can directly activate the expression of CBFs, thereby enhancing the plant’s cold resistance. In the early stage of cold stress, the kinase activity of BIN2 decreases, leading to the accumulation of unphosphorylated BZR1, which subsequently activates the expression of CBFs. In the later stage of cold stress, BIN2 phosphorylates ICE1 to promote the degraded by HOS1, thereby inhibiting its activation effect on CBFs genes. SL enhances cold resistance by triggering the degradation of WRKY41 protein mediated by MAX2 which is activated by cold. Additionally, SL promotes the degradation of SMXL, activating the expression of anthocyanin biosynthesis genes under cold stress. Cold signals interact with light signals. The expression of CBFs is inhibited by PIFs. Cold induces the degradation of EBF, allowing PIF3 to accumulate. Moreover, CRY2 stabilizes HY5, thereby allowing COR to be expressed during cold exposure. P, phosphorylation; Ub, ubiquitination.

BIN2 is a GSK3-like kinase in the brassinosteroid (BR) signaling pathway (Kim and Wang, 2010; Ryu et al., 2010). In the absence of BRs, active BIN2 phosphorylates transcription factors BRASSINAZOLE-RESISTANT 1 (BZR1) and BRI1-EMS-SUPPRESSOR 1 (BES1) to promote their degradation. Upon BR perception, BIN2 undergoes dephosphorylation by BRI1 SUPPRESSOR 1 (BSU1) and subsequent degradation via the 26S proteasome, leading to the release of BZR1 and BES1 from BIN2 inhibition (He et al., 2002; Wang et al., 2002). Dephosphorylated BZR1 and BES1 accumulate in the nucleus where they bind to target genes and induce BR responses (Wang et al., 2002; He et al., 2005; Sun et al., 2010). Continuous cold treatment induces the accumulation of dephosphorylated BZR1. Activated BZR1 induces *CBF1/2* by binding to conserved E-box/BRRE motifs in their promoters and can also directly regulate some *COR* genes independent of *CBFs*, such as *WRKY6*, *SAG21* and *SOC1*, to regulate the cold tolerance of plants (**Figure 2**).

In the early stage of cold stress, the activity of BIN2 kinase is suppressed while OST1 activity is stimulated. These two kinases work together to regulate the stability of ICE1, leading to upregulation in CBF expression. In the later stage, BIN2 activity increases, and the Ser94 of ICE1 is phosphorylated, which enhances their interaction. This results in ICE1 degradation through the 26S proteasome pathway, ultimately reducing *CBF* expression and preventing plants from mounting an excessive response to cold stress (**Figure 2**) (Ye et al., 2019). BIN2 also negatively modulates plant responses to cold stress by inhibiting low-temperature induced dephosphorylation of BZR1. However, some studies report that BIN2 phosphorylates AIF2, and enhances the stability of AIF2, which enhances ICE1 stability (Kim et al., 2024).

### 2.4 MAPK phosphorylation cascades exhibit species-specific functions in cold tolerance

The MAPK pathway is highly conserved across all eukaryotic organisms, serving as a crucial link between stimulus perception and cellular responses. The MAPK cascade consists of at least three protein kinases: MAPKKK/MEKK, MAPKK/MKK/MEK, and MAPK (MPK), which are activated in a sequential phosphorylation-dependent manner (Ichimura et al., 2002; Colcombet and Hirt, 2008). Indeed, emerging evidence indicates MAPK cascades play significant roles in modulating plant tolerance to cold stress (de Zelicourt et al., 2016).

In *Arabidopsis*, constitutive activation of the MKK4/5-MPK3/6 signaling cascade in plants phosphorylates ICE1 at Ser94, Ser203, Thr366, Thr382, Thr384, and Ser403, which promotes ICE1 degradation and thereby negatively regulates *CBF* expression (Zhao et al., 2017). Similarly, phosphorylation of ICE1 by MPK3 and MPK6 in maize (*Zea mays*) decreases its stability and transcriptional activity, thereby negatively regulating *CBF* expression and plant cold responses (Jiang et al., 2022a). Under cold stress, ZmMPK8 phosphorylates bZIP68 at its Ser250 site to promote bZIP68 stability and DNA-binding affinity, which enhances transcriptional inhibition of *COR* genes (such as DREB1s), especially in the later stage of cold stress response (Li et al., 2022; Gao et al., 2024). Meanwhile, ZmMPK8 phosphorylates the A-type response regulator ZmRR1, promoting its degradation via the 26S proteasome pathway. This downregulation of ZmRR1 results in reduced expression of *ZmDREB1* and *CELLULOSE SYNTHASE A* (*CesA*) genes, thereby negatively regulating the cold tolerance of maize (Zeng et al., 2021).

Conversely, accumulating evidence indicates that the MEKK1-MKK2-MPK4/6-mediated MAPK signaling cascade can constitutively suppress MPK3 and MPK6 activity, playing a positive regulatory role in low temperature responses (Teige et al., 2004). In rice, cold stress enhances the kinase activity of OsMAPK3. OsMAPK3 phosphorylates OsbHLH002/OsICE1 and disrupts the interaction between OsbHLH002/OsICE1 and the E3 ubiquitin ligase OsHOS1, which stabilizes OsbHLH002/OsICE1. OsbHLH002/OsICE1 directly activates the transcription of *OsTPP1* which encodes a key enzyme for trehalose biosynthesis, thereby increasing the levels of trehalose to enhance the cold resistance of rice (Zhang et al., 2017). This functional divergence suggests that the ICE1-CBF module, while structurally conserved across angiosperms, has been rewired differently in various lineages to meet distinct ecological and physiological demands. Notably, the species-specific outcome of MAPK-mediated ICE1 regulation correlates with differences in the surrounding regulatory network: in *Arabidopsis*, multiple negative regulators (MPK3/6, BIN2, HOS1) converge on ICE1, whereas in rice, the regulatory balance appears skewed toward positive modulation by MAPK3. These observations underscore the importance of investigating PTM regulatory networks in a wider range of crop species beyond model plants, as insights derived from *Arabidopsis* are not universally transferable to agronomically important crops.

Collectively, phosphorylation regulates cold tolerance through four principal mechanisms. First, it directly modulates the stability of key regulatory proteins: OST1-mediated phosphorylation stabilizes ICE1, whereas BIN2- and MPK3/6-mediated phosphorylation promotes ICE1 degradation. Second, phosphorylation controls the subcellular localization of transcription factors, as exemplified by CPK28-mediated phosphorylation of NLP7, which triggers its translocation from the cytoplasm to the nucleus. Third, phosphorylation serves as a hub for signal integration, where multiple kinases (OST1, BIN2, MAPKs) converge on ICE1 to integrate inputs from ABA, BR, and stress-activated pathways. Fourth, phosphorylation exhibits temporal dynamics, as seen in the early activation of OST1 versus the late-stage activation of BIN2, enabling plants to fine-tune cold responses over time. These mechanistic principles not only explain how individual phosphorylation events contribute to cold tolerance but also provide a framework for understanding their integration with other PTMs.

## 3 Ubiquitination

Ubiquitination is a post-translational modification of conserved proteins in plants, playing an essential role in the responses to abiotic stress (Hellmann and Estelle, 2002; Chen and Sun, 2009; Sharma et al., 2021). Ubiquitination is a multi-step process in which ubiquitin (Ub) is conjugated to a target protein through sequential reactions catalyzed by three enzymes: E1 ubiquitin-activating enzyme, E2 ubiquitin-conjugating enzyme, and E3 ubiquitin ligase. Typically, E3 ligases specifically recognize target proteins (Vierstra, 2009; Sadanandom et al., 2012). Ubiquitin is a highly conserved protein containing 76 amino acid residues, and the linkage between the target and ubiquitin occurs through seven lysine residues (K6, K11, K27, K29, K33, K48, and K63) (Bhat and Greer, 2011; Komander and Rape, 2012). Based on their structural features, E3 ubiquitin ligases are classified into single-subunit types, which include the RING, U-box, and HECT families, and multi-subunit complexes, such as the SCF (SKP1-CUL1-F-box) and APC/C (Anaphase Promoting Complex/Cyclosome) complexes (Lee and Kim, 2011).

### 3.1 Single-subunit E3 ubiquitin ligases in cold signaling

#### 3.1.1 RING-type single-subunit E3 ligases in cold signaling

The RING-type E3 ligases constitute one of the largest families of single-subunit E3 ligases and play diverse roles in plant cold responses. RING-type E3 ligases including HOS1, MaSEVEN IN ABSENTIA1 (MaSINA), *Arabidopsis* ARABIDOPSIS TOXICOS EN LEVADURA 78 (AtATL78), CONSTITUTIVE PHOTOMORPHOGENIC 1 (COP1), MYB30-INTERACT-ING E3 LIGASE1 (MdMIEL1) have been identified as significant regulators of plant responses to cold stress (**Table 1**). As described above, HOS1 facilitates ICE1 degradation and thus exerts a negative regulatory effect on cold resistance (**Figure 1**). Similar to HOS1, the banana RING-type E3 ligase MaSINA1 also interacts with MaICE1, leading to its ubiquitination and subsequent degradation, thereby negatively regulating cold responses in bananas (Fan et al., 2017). AtATL78 was found to be upregulated in response to cold stress and functions as a negative regulator; however, its target gene remains unknown (Yan et al., 2003). In rice, OsATL38 negatively modulates the cold tolerance of rice through ubiquitinating OsGF14d, a 14-3–3 protein of rice within the cold signaling pathway (Cui et al., 2022). COP1 functions as a master repressor of photomorphogenesis. Under cold stress, phosphorylated CRY2 (induced by blue light) is stabilized and interacts with COP1, which inhibits the interaction between COP1 and HY5 and subsequently reduces the degradation of HY5. Accumulated HY5 directly activates the expression of B-BOX DOMAIN PROTEIN7 (BBX7) and BBX8, which positively regulates freezing tolerance by controlling the expression of a set of COR genes, including anthocyanin biosynthesis genes, through a CBF-independent pathway (**Figure 2**) (Li et al., 2021). In apple, the RING-type E3 ligase MdMIEL1 interacts with MdBBX37 to promote its ubiquitination and degradation, thereby negatively regulating cold tolerance (An et al., 2021).

#### 3.1.2 U-box type E3 ubiquitin ligases regulate plant cold adaptation by ubiquitinating ICE1 and MYB15

Two U-box type E3 ubiquitin ligases, PUB25 and PUB26 primarily facilitate the K63-linked ubiquitination of ICE1 during the early stages of cold stress response, thereby stabilizing ICE1 while promoting degradation of MYB15. Consequently, stabilized ICE1 directly represses the transcriptional activity of MYB15, leading to significant induction of *CBF* gene expression. During prolonged cold stress (late stages), K48-linked ubiquitin chains on ICE1 accumulate, resulting in its degradation, which allows MYB15 to restore its protein abundance. Consequently, the initial cold-induced increase in CBF transcript levels begins to decline (**Figure 1**) (Wang et al., 2019, 2023b).

### 3.2 SCF-type multi-subunit E3 complexes in cold signaling

In contrast to single-subunit RING-and U-box-type E3 ligases, the SCF (SKP1-CUL1-F-box) complexes are multi-subunit E3 ligases in which F-box proteins function as substrate-recognition components (Lee and Kim, 2011). Several F-box proteins have been identified as key regulators of cold tolerance. The F-box proteins EIN3 BINDING F-BOX1 (EBF1) and EBF2, as substrate-recognition components, assemble with the core complex containing SKP1, Cullin 1, and RBX1 to form functional SCF E3 complexes. They directly target PHYTOCHROME-INTERACTING FACTOR 3 (PIF3) for degradation via the 26S proteasome pathway (Guo and Ecker, 2003; Potuschak et al., 2003). At a temperature of 22 °C, light induces PIF3 degradation by EBF1/2, while darkness leads to EBF1/2 degradation and PIF3 stabilization. Under cold stress at 4 °C, the EBF1/2 proteins is degraded regardless of light conditions, resulting in increased stability of the PIF3. PIF3 is able to directly bind to the promoter of CBF genes and inhibit their expression (Jiang et al., 2017), leading to cold sensitivity (**Figure 2**).

The F-box protein MORE AXILLARY GROWTH2 (MAX2) is a component of the SCF complex that modulates the degradation of SUPPRESSOR OF MAX2 1/SMAX1-like (SMAX1/SMXL) family members through the ubiquitin 26S proteasome pathway in the strigolactones (SL) signaling (Yang et al., 2019; Khosla et al., 2020). SL promotes the interaction between MAX2 and WRKY41, mediates the degradation of WRKY41, relieves WRKY41-mediated inhibition of *CBF* and their downstream genes, and thus enhances cold tolerance (Zheng et al., 2023) (**Figure 2**).

In apple, jasmonic acid (JA) signaling also engages SCF-type ubiquitination to modulate cold tolerance. In the absence of JA, MdJAZ1/2 interact with MdBBX37, which weakens the transcriptional activation activity of MdBBX37 on the promoters of MdCBF1 and MdCBF4, disrupts the MdBBX37-MdICE1 complex, and thus reduces cold tolerance. In the presence of JA, MdJAZ1/2 are recognized by the JA receptor COI1-an F-box protein-and degraded via the SCF-type E3 complex. This releases MdBBX37, which then directly activates MdCBF1 and MdCBF4 expression and forms a complex with MdICE1 to co-activate MdCBF1, enhancing cold tolerance (An et al., 2021). (Note: MdBBX37 is a transcription factor, not an E3 ligase; the SCF-type E3 complex containing COI1 mediates the ubiquitination and degradation of MdJAZs.).

Ubiquitination regulates cold tolerance through distinct modes of action depending on the type of ubiquitin linkage and the identity of the E3 ligase. K48-linked polyubiquitination generally targets proteins for proteasomal degradation, as exemplified by HOS1-mediated degradation of ICE1. In contrast, K63-linked ubiquitination often confers non-proteolytic regulatory functions, including protein stabilization, as demonstrated by PUB25/26-catalyzed K63-linked ubiquitination of ICE1 during the initial phase of cold stress. Furthermore, E3 ligases act at different levels of the signaling hierarchy: HOS1 and PUB25/26 directly target the master transcription factor ICE1, while SCF-type complexes (EBF1/2, MAX2, COI1) regulate transcriptional repressors (PIF3, WRKY41, JAZs) that indirectly modulate CBF expression. This multi-level regulation allows ubiquitination to finely tune cold responses at both transcriptional and post-translational levels.

## 4 Other post-translational modifications

Beyond phosphorylation and ubiquitination, emerging PTMs including SUMOylation, acetylation, lysine crotonylation, and S-acylation have been increasingly recognized as critical regulators of cold stress responses. Although these PTMs have traditionally been studied in isolation, recent large-scale PTM profiling-including sumo-proteomics, acetylomics, and crotonylation analyses-has substantially expanded our understanding of their scope and potential functions.

### 4.1 SUMOylation

Small Ubiquitin-like Modifier (SUMO) is a small polypeptide that modifies other proteins through isopeptide bonding. This ubiquitin-like protein modification is involved in the regulation of many processes, including plant responses to stress, pathogen defense, ABA signaling, and floral induction. SUMO modification can either shield protein surfaces to disrupt protein interactions or create new interaction interfaces to promote alternative protein assemblies (Hermkes et al., 2011). The SUMO E3 ligase SIZ can catalyze SUMOylation of ICE1 at the K393, which inhibits the ubiquitination by HOS1 and thus promotes the stability of ICE1. This results in the up-regulation of *CBF3/DREB1A* expression and the inhibition of the cold-negative regulator *MYB15* expression, making the plant more cold-resistant (**Figure 1**) (Miura et al., 2007). The degradation of ICE1 depends on the ubiquitination mediated by the E3 ubiquitin ligase HOS1 (Dong et al., 2006), while SUMOylation maintains the stability of ICE1 by suppressing its ubiquitination. Notably, BIN2 phosphorylates ICE1, thereby promoting its physical interaction with HOS1 and accelerating its proteasomal degradation (Ye et al., 2019); however, the functional crosstalk between phosphorylation and SUMOylation on ICE1 remains unclear.

In apple, cold stress increases the expression of the transcription factor MdMYB2, which binds to the *MdSIZ1* promoter and activates its transcription, contributing to increased cold tolerance (Zhou et al., 2017; Jiang et al., 2022b). Furthermore, the ARABIDOPSIS THALIANA RESPONSE FACTOR 1 (ARR1) serves as an important component of the cytokinin signaling pathway. Under cold stress conditions, ARR1 undergoes phosphorylation and then is transported to the nucleus where it binds to chromatin and interacts with the SUMO E3 ligase MMS21 (HPY2), promoting its SUMOylation. This, in turn, enhances the acetylation of histone H3, thereby activating the ARR1-dependent transcription in response to cold stress (Kang et al., 2024). These findings demonstrate that SUMOylation can modulate diverse transcription factors to promote cold adaptation.

### 4.2 Acetylation

Protein acetylation is a reversible post-translational modification mediated by acetyltransferases and deacetylases, which directly alters the binding capacity and function of proteins by changing the physicochemical properties and conformation of proteins. Acetylation is a mechanism by which cells regulate gene expression and physiological processes. HOS15 binds to and degrades HISTONE DEACETYLASE 2C (HD2C) under cold stress, resulting in the increase of histone acetylation level on the promoter of *COR* gene. This makes it easier for *CBF* to bind to the promoter of *COR*. At the same time, HOS15 can also interact with *CBF*, further promoting the binding of CBF protein to the promoter of *COR*, and thereby accelerating the expression of *COR* gene in response to cold stress (Park et al., 2018). Histone deacetylase (HDAC) OsHDA716 mediates deacetylation of the transcription factor OsbZIP46, which reduces OsbZIP46 transcriptional activity and protein stability. This ultimately impairs cold-induced Ca^2+^ influx and reduces the cold tolerance of rice (Sun et al., 2024).

### 4.3 Lysine crotonylation

Lysine crotonylation is a recently identified PTM in plants. It has been identified in the protein LIPOCALIN-1-LIKE 1(DgTIL1) from chrysanthemum. DgTIL1 interacts with a NON-SPECIFIC PLASMA LIPID TRANSFER PROTEIN (DgnsLTP), which promotes the expression and activity of *PEROXIDASE* (*POD*). This reduces the accumulation of reactive oxygen species (ROS) and improves tolerance of chrysanthemum to low-temperature stress. Low temperature increases the crotonylation level of DgTIL1 at Lys72, which prevents DgnsLTP degradation, further reducing ROS levels and enhancing cold resistance (Huang et al., 2021). In addition, the crotonylation level of GLUTATHIONE PEROXIDASE 1 (GPX1) at Lys220 is decreased at low temperature, which increases DgGPX1 activity, reduces ROS accumulation, and enhances cold resistance in chrysanthemum (Yang et al., 2022).

### 4.4 S-acylation

S-acylation, commonly referred to as S-palmitoylation, is a crucial lipidation process for proteins in eukaryotic cells. Unlike other forms of protein lipidation such as myristoylation and prenylation, S-acylation is reversible. The protein S-acyltransferases (PATs) catalyze the S-acylation of proteins. In contrast, acyl protein thioesterases (APTs) are responsible for mediating the de-S-acylation of proteins (Lanyon-Hogg et al., 2017). In *Medicago truncatula*, MtNAC80 is localized to the plasma membrane through S-acylation by MtPAT9. Under cold stress, MtNAC80 is transported to the nucleus by de-S-acylation mediated by thioesterases such as MtAPT1. In the nucleus, MtNAC80 binds to the promoter of glutathione S-transferase *MtGSTU1* and activates its expression. MtGSTU1 removes excessive H_2_O_2_ and malondialdehyde, enabling plants to survive cold stress (Ye et al., 2024).

## 5 Conserved and species-specific PTM regulatory mechanisms

A central question in plant cold biology is the extent to which PTM-mediated regulatory mechanisms identified in model species are conserved across diverse crop plants. Comparative analysis reveals both evolutionary conservation and lineage-specific divergence, reflecting the distinct ecological niches and adaptive strategies of different species.

The ICE1-CBF-COR pathway exemplifies this dichotomy. ICE1 exhibits functional conservation across angiosperms, and its overexpression enhances cold tolerance in tomato, cucumber and *Arabidopsis*. Moreover, the antagonistic interplay between HOS1-mediated ubiquitination and SIZ1-mediated SUMOylation at the ICE1 regulatory node is conserved in *Arabidopsis*, rice, and banana (Dong et al., 2006; Fan et al., 2017; Zhang et al., 2017). These findings suggest that the ubiquitination-SUMOylation balance on ICE1 is an evolutionarily ancient regulatory mechanism for fine-tuning cold responses.

**Figure 3:**
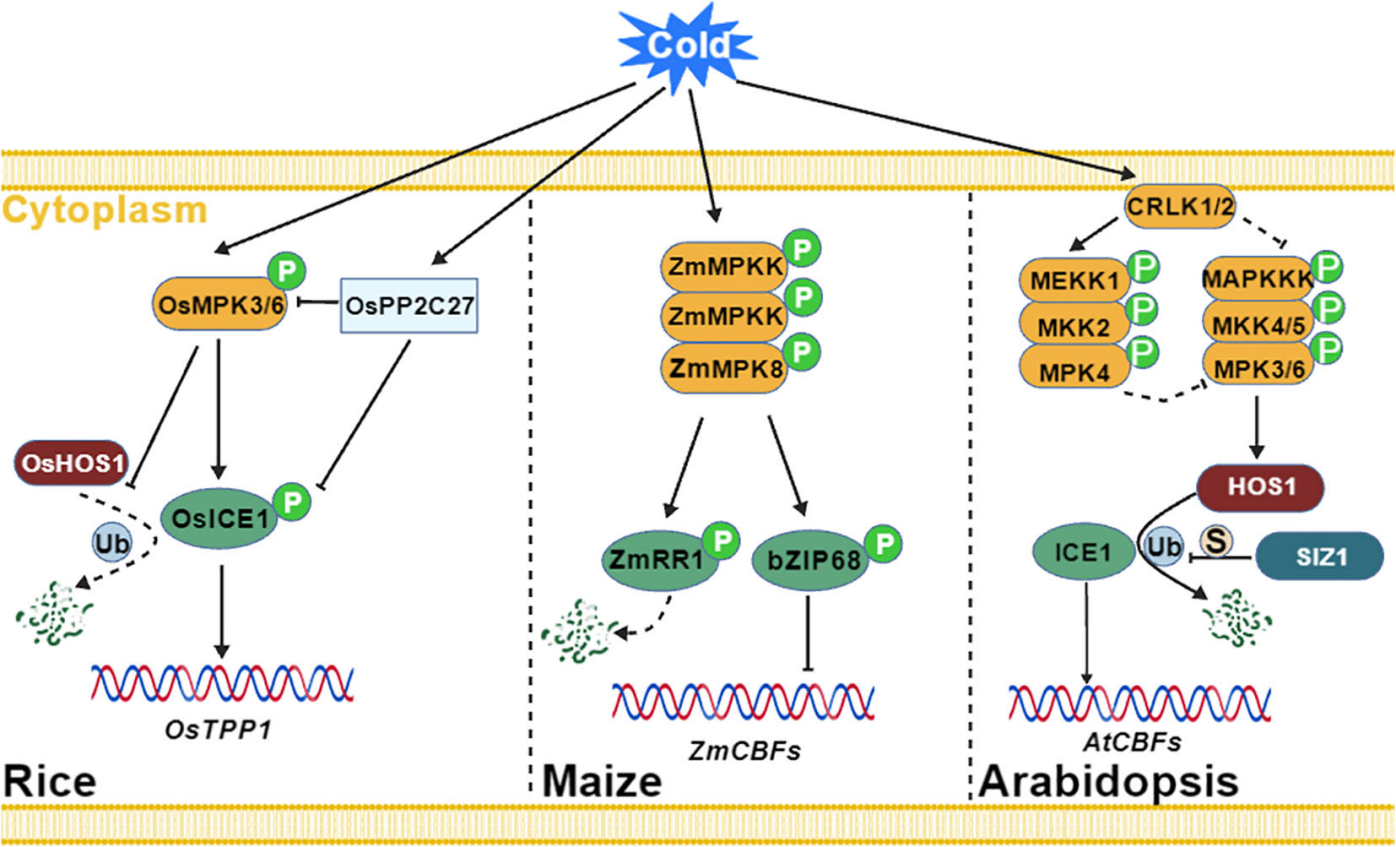
Specific regulation of the MAPK cascade signaling pathway under cold stress conditions in different species. In rice, cold stimuli trigger autophosphorylation of OsMPK3/6. The activated OsMPK3/6 further phosphorylates OsICE1 to drive the transcription of cold-responsive gene OsTPP1. Meanwhile, OsMPK3/6 can repress the activity of E3 ubiquitin ligase OsHOS1. This blocks OsHOS1-mediated ubiquitination and proteasomal breakdown of OsICE1, ultimately strengthening rice cold tolerance. By contrast, the phosphatase OsPP2C27 serves as a negative brake for cold signaling: it hinders OsMPK3/6 autophosphorylation and dephosphorylates phosphorylated OsICE1 to dampen downstream cold responses. Phosphorylated ZmMPK8 exerts dual inhibitory effects on cold resistance. It phosphorylates positive regulator ZmRR1 and accelerates its degradation; on the other hand, phosphorylation of negative repressor bZIP68 by ZmMPK8 stabilizes this protein and improves its capacity to bind target DNA. Together, these two events suppress the expression of ZmCBFs genes and compromise maize tolerance to low temperature. In *Arabidopsis*, plasma membrane-localized CRLK1 and CRLK2 kick off two parallel MAPK phosphorylation cascades: the MEKK1-MKK2-MPK4 module and the MAPKKK-MKK4/5-MPK3/6 module. Once activated, MPK3/6 phosphorylates ICE1. This modification makes ICE1 more susceptible to HOS1-dependent ubiquitination and subsequent proteasome degradation, which weakens the plant’s ability to withstand cold stress. P, phosphorylation; Ub, ubiquitination; S, SUMOylation.

However, the functional consequences of MAPK-mediated ICE1 phosphorylation display striking species-specific variation. In *Arabidopsis* and maize, phosphorylation of ICE1 by MPK3/6 (in *Arabidopsis*) or ZmMPK8 (in maize) promotes its proteasomal degradation, thereby negatively regulating cold tolerance (Zhao et al., 2017; Jiang et al., 2022a; Zeng et al., 2021). In contrast, in rice, OsMAPK3 phosphorylates OsbHLH002/OsICE1 to enhance its stability and transcriptional activity, thereby positively modulating cold acclimation (Zhang et al., 2017). Cold stress triggers the transcriptional induction of OsPP2C27. This phosphatase dephosphorylates activated OsMAPK3 to dampen its kinase activity, and also directly dephosphorylates OsbHLH002/OsICE1, facilitating its subsequent degradation (Xia et al., 2021). This dual-target dephosphorylation strategy enables rapid and flexible attenuation of the OsMAPK3- OsbHLH002/OsICE1-OsTPP1 signaling cascade. This mechanism avoids persistent overactivation of cold response signaling and allows plants to resume regular growth once cold signals subside (Xia et al., 2021). (**Figure 3**). This functional divergence suggests that the surrounding regulatory network has been rewired differently during evolution. Similarly, in maize, the cold-responsive bHLH transcription factor COOL1, which is targeted by ZmCPK17 phosphorylation, has no clear ortholog in *Arabidopsis*, highlighting species-specific regulatory nodes (Zeng et al., 2025).

Calcium signaling also reveals both conservation and divergence. Cold-induced Ca^2+^ influx is a universal early response across species, yet the downstream calcium sensors differ: in *Arabidopsis*, the CPK28-NLP7 module functions as a central regulatory axis (Ding et al., 2022), whereas ZmCPK17-COOL1 fulfills analogous functions in maize (Zeng et al., 2025). These differences suggest that while the general framework of Ca^2+^-PTM signaling is conserved, the specific sensor-substrate pairs have diversified to meet distinct physiological demands.

Another layer of complexity arises from recent large-scale PTM profiling across species. Evolutionary conservation of PTM sites frequently correlates with functional significance: phosphorylation, ubiquitination, and S-acylation are preferentially enriched in highly conserved protein regions. Comparative phosphoproteomics between *Arabidopsis* and rice has identified both shared and species-specific cold-responsive phosphosites, providing a systems-level view of regulatory divergence.

Understanding these conserved and divergent PTM regulatory modules holds not only fundamental biological significance but also substantial translational potential. Conserved PTM regulatory nodes-such as the ICE1-ubiquitination/SUMOylation balance-represent promising targets for engineering broad-spectrum cold tolerance across multiple crops. Conversely, species-specific regulators, such as the COOL1 module in maize or the MAPK signaling variants, could be leveraged for tailored improvements in individual crops. This highlights the importance of validating PTM-based findings from *Arabidopsis* in diverse crop species through comparative PTM studies, facilitated by advances in pan-genomics and cross-species proteomics.

## 6 PTM crosstalk in cold stress signaling

In plant cold stress responses, different PTMs do not function in isolation; rather, they form an intricate regulatory network through interconnected crosstalk. This interplay allows plants to exert precise spatiotemporal control of cold signaling, integrating multiple layers of regulation to finely balance between stress tolerance and growth.

Among the most well-characterized examples is the antagonistic crosstalk between ubiquitination and SUMOylation at the ICE1-CBF regulatory hub. The E3 ubiquitin ligase HOS1 mediates K48-linked ubiquitination of ICE1, promoting its degradation via the 26S proteasome and thereby attenuating cold responses (Dong et al., 2006). Conversely, the SUMO E3 ligase SIZ1 mediates SUMOylation of ICE1 at Lys393, which competes with ubiquitination at the same or adjacent lysine residues. SUMOylation physically protects ICE1 from HOS1-mediated ubiquitination, thereby stabilizing ICE1 and enhancing CBF expression (Miura et al., 2007). This reciprocal regulation-where phosphorylation by BIN2 enhances ICE1-HOS1 interaction and promotes ICE1 degradation (Ye et al., 2019), while SUMOylation antagonizes this process-reveals a multi-layered regulatory circuit that tightly controls the amplitude and duration of cold signaling (**Figure 1**).

Phosphorylation also plays a dual role in regulating ubiquitination-dependent protein turnover. OST1-mediated phosphorylation of ICE1 at multiple serine residues not only enhances its transcriptional activity but also inhibits its interaction with HOS1, thereby shielding ICE1 from ubiquitination and proteasomal degradation (Ding et al., 2015). Similarly, OST1 phosphorylates PUB25 and PUB26 to promote their E3 ligase activity, which in turn ubiquitinates and degrades the transcriptional repressor MYB15, relieving its inhibition of *CBF* expression (Wang et al., 2019, 2023b). In the MAPK cascade, MPK3/6-mediated phosphorylation of ICE1 at Ser94, Ser203, and other residues promotes its degradation, counteracting the stabilizing effect of OST1-mediated phosphorylation at different sites (Zhao et al., 2017) (**Figure 1**). This site-specific phosphorylation code-where distinct kinases phosphorylate different residues of the same protein to elicit opposing outcomes-represents a sophisticated mechanism for integrating diverse environmental signals into coordinated cold responses.

Crosstalk also extends to epigenetic regulation via acetylation. Under cold stress, HOS15 mediates the degradation of the histone deacetylase HD2C, increasing histone acetylation levels at *COR* gene promoters and enhancing chromatin accessibility for CBF-mediated transcriptional activation (Park et al., 2018). This suggests that ubiquitination can indirectly modulate chromatin remodeling by targeting histone deacetylases, providing a link between PTM-mediated signaling and epigenetic reprogramming during cold acclimation.

In addition, emerging evidence highlights intricate the crosstalk between SUMOylation and other PTMs. For instance, the cytokinin response factor ARR1 is phosphorylated in response to cold stress, triggering its nuclear translocation; subsequently, it undergoes SUMOylation mediated by the E3 ligase MMS21. This SUMOylation event promotes histone H3 acetylation and facilitates ARR1-dependent transcriptional activation (Kang et al., 2024). This sequential phosphorylation-SUMOylation-acetylation cascade illustrates how multiple PTMs can be temporally coordinated to achieve full transcriptional activation.

Collectively, these examples demonstrate that PTM crosstalk operates at multiple levels: from competition for modification sites on the same protein, to hierarchical cascades where one modification primes another, to indirect regulation through effector proteins. Unraveling this complex interplay is essential for understanding how plants achieve signaling specificity and dynamic responsiveness in their adaptation to cold stress.

## 7 Conclusion and perspectives

PTMs function as essential molecular switches and regulatory nodes in plant responses to cold stress, integrating signal perception, transduction, and downstream gene expression. In *Arabidopsis thaliana*, PTMs including phosphorylation, ubiquitination, SUMOylation, and acetylation constitute a highly interconnected signaling network that coordinately regulates responses to various stages of cold stress (**Figure 1**). Nevertheless, several critical questions remain unresolved, and addressing them will require both technological innovation and a shift in research paradigms.

First, while phosphorylation is the most extensively studied PTM, current research predominantly focuses on kinases, with relatively limited identification and characterization of corresponding phosphatases. Moreover, the phosphorylation of different sites of the same protein, which can be mediated by a single kinase or distinct kinases, even triggers opposing responses to cold stress. This site-specific functional divergence poses a significant challenge in understanding how phosphorylation states are precisely coordinated. Future work should therefore prioritize the systematic identification of phosphatases and the development of phosphosite-specific antibodies or phospho-mimetic/mutant lines to dissect the distinct functions of individual phosphorylation events. Second, Ubiquitination and phosphorylation often occur concurrently: phosphorylation of substrates can influence their ubiquitination status, and in some cases, phosphorylation directly targets E3 ubiquitin ligases, modulating their enzymatic activity. Nevertheless, it remains unclear whether kinases and phosphatases themselves undergo ubiquitination or are subject to ubiquitin-mediated regulation. Deciphering this reciprocal regulation will require integrated proteomic approaches that simultaneously resolve the dynamics of phosphorylation and ubiquitination. Third, research on SUMOylation and acetylation is still in their early stages relative to cold stress signaling, representing a promising frontier for future investigations.

Beyond these PTM-specific questions, a major emerging challenge is to understand the spatial and temporal dynamics of PTM crosstalk. Cold stress triggers a cascade of events from perception at the plasma membrane to nuclear transcriptional reprogramming, yet most current studies provide only static snapshots of PTM status at single time points. How phosphorylation, ubiquitination, and SUMOylation sequentially coordinate their actions across different cellular compartments during cold acclimation remains largely unexplored. Advances in spatiotemporally resolved proteomics and biosensor technologies will be essential to capture these dynamic processes.

Plant responses to cold stress are also influenced by other environmental and phytohormone signals, such as light signals, jasmonic acid, ABA, and strigolactone. Under cold stress conditions, components in these signaling pathways undergo PTMs, which regulate cold responses in a CBF-dependent or independent manner. This suggests that PTMs play important roles in the crosstalk between cold stress and other signals, and it represents an interesting and important research direction for the future. Unraveling how PTMs integrate multiple stress and hormone signals will require multi-signaling network modeling.

In recent years, research on PTMs in the cold stress signaling pathways of many crops and ornamental has increased. For example, phosphorylation plays an important role in the cold stress responses of rice and maize; ubiquitination is involved in cold resistance in bananas, rice, apples and tomatoes; SUMOylation and acetylation regulate the cold tolerance of bananas and chrysanthemums. This indicates that PTMs have potential applications in cold tolerance breeding of crops and ornamental plants. However, most studies have focused on the functional verification of PTMs of a few reported genes (such as *OsICE1* in rice, *MdCBF1/2* in apples, and *SlICE1* in tomatoes), and mostly adopted the “homologous verification” approach in model plants, lacking systematic understanding of the natural PTM site polymorphisms in crop germplasm resources. In fact, the resistance of many cold-resistant germplasm resources does not originate from coding region mutations, but from the functional polymorphisms of regulatory regions or PTM sites. In the future, large-scale germplasm resources should be utilized to conduct natural variation screening of PTM sites. We recommend that future efforts focus on germplasm-scale natural variation screening of PTM sites combined with multi-omics and GWAS analyses. This integrative strategy will facilitate the identification of elite haplotypes associated with beneficial PTM patterns, thereby bridging mechanistic insights into PTMs with actionable breeding applications.

The integration of PTM knowledge with CRISPR/Cas9-based genome editing has enabled the genetic validation and functional exploitation of key regulators in cold signaling pathways. In rice, CRISPR/Cas9-mediated knockout of *OsMYB30*, a transcription factor that negatively regulates β-amylase expression by forming a complex with *OsJAZ9* under cold stress, significantly enhanced cold tolerance by affecting starch degradation and maltose accumulation (Zeng et al., 2020). Notably, multiplex CRISPR/Cas9 editing simultaneously targeting *OsPIN5b*, *GS3*, and *OsMYB30* not only improved cold tolerance but also increased grain yield, thereby demonstrating the feasibility of pyramiding multiple agronomically valuable traits in a single editing event (Zeng et al., 2020). Conversely, CRISPR/Cas9 knockout of positive regulators such as *OsAnn3*, *OsAnn5*, and *OsPRP1* in rice resulted in increased cold sensitivity, confirming their protective roles against chilling injury (Shen et al., 2017). In tomato, CRISPR/Cas9-generated *cbf1* mutants exhibited enhanced chilling damage with increased electrolyte leakage and higher accumulation of reactive oxygen species, indicating that *CBF1* is essential for cold acclimation (Li et al., 2018). Similarly, CRISPR/Cas9-mediated knockout of *FvICE1* in strawberry led to reduced cold and drought tolerance, consistent with its role as a positive regulator of stress responses (Han et al., 2023). Furthermore, CRISPR/Cas9 can be integrated with PTM mechanistic studies to generate precise phospho-mimetic or degradation-resistant alleles through base editing or homology-directed repair, enabling functional interrogation of individual PTM events in their native genomic context-an approach that holds particular promise for engineering cold-tolerant crops by fine-tuning the activity and stability of master regulators such as *ICE1*, *CBFs*, and *OST1* (Kumar et al., 2023). However, the successful translation of these strategies from model plants to economically important legume crops remains challenging due to limitations in transformation efficiency, low regeneration rates, and genotype dependency (Kumari et al., 2025). Recent advances in optimizing tissue culture protocols, exploring alternative explants, and improving DNA delivery methods are progressively addressing these bottlenecks, offering new opportunities for applying CRISPR-based genome editing to enhance abiotic stress tolerance in pulses (Kumari et al., 2025). Collectively, these studies illustrate how CRISPR/Cas9-mediated mutagenesis provides a powerful platform for functional validation of cold signaling components and their PTM-regulated networks, thereby bridging mechanistic insights with translational breeding applications.

In conclusion, studies on PTM regulation in plant cold signaling stand at a crucial transition stage. Moving beyond descriptive identification of PTM events, in-depth exploration of PTM dynamics, crosstalk and natural variation will deepen our understanding of plant cold adaptation and unlock new avenues for crop genetic improvement. The combination of advanced proteomics, CRISPR editing and germplasm screening will bridge basic PTM research and practical breeding, facilitating the creation of climate-resilient crops.

While the majority of current knowledge on PTM-mediated cold signaling derives from gene-by-gene functional studies, recent advances in mass spectrometry-based proteomics have enabled large-scale profiling of PTM dynamics under cold stress, providing a systems-level view of the regulatory landscape. Phosphoproteomic analyses in *Arabidopsis* and rice have identified hundreds of cold-responsive phosphorylation events, revealing not only known regulators (e.g., OST1, MAPKs, and CPKs) but also numerous previously uncharacterized kinases and phosphosites that represent new avenues for functional investigation (Liu et al., 2021; Chen et al., 2021). Similarly, ubiquitinomics has been applied to profile the ubiquitinated proteome under low-temperature conditions, identifying potential substrates of cold-responsive E3 ligases and shedding light on the extent of ubiquitination-mediated regulation beyond the well-studied ICE1-CBF axis.

SUMOylation, acetylation, and crotonylation have also been subjected to global profiling using specific enrichment strategies. Sumo-proteomic approaches have identified hundreds of SUMO targets in *Arabidopsis* under stress conditions, many of which are transcription factors and chromatin remodelers. Acetylomics has revealed widespread acetylation of histones and non-histone proteins in response to cold, linking PTMs to epigenetic regulation. Crotonylation profiling, though still in its infancy, has identified DgTIL1 as a target in chrysanthemum (Huang et al., 2021) and GLUTATHIONE PEROXIDASE 1 (GPX1) as an additional crotonylated protein (Yang et al., 2022), suggesting that this modification may be more prevalent and functionally significant than currently recognized.

The integration of multi-omics data-combining transcriptomics, proteomics, and multiple PTM profiles-holds particular promise for dissecting PTM crosstalk. For instance, simultaneous profiling of phosphorylation and ubiquitination from the same sample can reveal dynamic relationships between these two modifications on the same proteins, such as the phosphorylation-dependent ubiquitination observed for ICE1 and PUB25/26. However, there are still several challenges: certain PTMs are relatively low in abundance, and extensive sample enrichment is required; the dynamic range of PTM changes under cold stress can be narrow; and the functional validation of large-scale datasets remains a bottleneck. Despite these challenges, the continued development of PTM-enrichment strategies, quantitative proteomics, and computational integration tools promises to transform our understanding from linear signaling pathways to complex, interconnected PTM networks that coordinately regulate plant cold adaptation.
